# The ED_50_ and ED_95_ of oxytocin infusion rate for maintaining uterine tone during elective caesarean delivery: a dose-finding study

**DOI:** 10.1186/s12884-019-2692-x

**Published:** 2019-12-31

**Authors:** Xiao Wei Qian, Dan M. Drzymalski, Chang Cheng Lv, Fei He Guo, Lu Yang Wang, Xin Zhong Chen

**Affiliations:** 1 Department of Anesthesia, Women’s Hospital, Zhejiang University School of Medicine, Xueshi Road 1, Hangzhou, 310006 China; 2 Department of Anesthesiology and Perioperative Medicine, Tufts Medical Center, Boston, MA USA

**Keywords:** Caesarean section, Oxytocin, Postpartum haemorrhage prevention

## Abstract

**Background:**

The 90% effective dose (ED_90_) of oxytocin infusion has been previously estimated to be 16.2 IU h^− 1^. However, bolus administration of oxytocin prior to the infusion may decrease the infusion dose required. The aim of this study was to estimate the ED_95_ for oxytocin infusion after a bolus at elective caesarean delivery (CD) in nonlaboring parturients.

**Methods:**

We performed a randomized, triple blinded study in 150 healthy termparturients scheduled for elective CD under epidural anaesthesia. After delivery of the infant and i.v. administration of 1 IU oxytocin as a bolus, Participants were randomized to receive oxytocin infusion at a rate of 0, 1, 2, 3, 5, or 8 IU h^− 1^, to be given for a total of 1 h. Uterine tone assessed by the blinded obstetrician as either adequate or inadequate. Secondary outcomes included estimated blood loss (EBL), requirement for supplemental uterotonic agents, and development of side effects.

**Results:**

The 95% effective dose (ED_95_) of oxytocin infusion was estimated to be 7.72 IU h^− 1^ (95% confidence interval 5.80–12.67 IU h^− 1^). With increasing oxytocin infusion rate, the proportion of parturients who needed rescue oxytocin bolus or secondary uterotonic agents decreased. No significant among-group differences in the EBL and oxytocin-related side effects were observed.

**Conclusions:**

In parturients who receive a 1 IU bolus of oxytocin during elective cesarean delivery, an infusion rate of oxytocin at 7.72 IU h^− 1^ will produce adequate uterine tone in 95% of parturients. These results suggest that the total dose of oxytocin administered in the postpartum period can be decreased when administered as an infusion after oxytocin bolus.

## Background

Although oxytocin has been administered for postpartum haemorrhage prophylaxis for several decades [1], there still remains considerable variability in the approach to its administration during caesarean delivery (CD). As an example, a slow i.v. bolus dose of up to 5 IU has been advocated by several authors [2, 3], but Carvalho et al. estimated the minimum effective dose (ED) of oxytocin bolus to result in adequate tone in 90% of parturients (ED_90_) during elective CD in nonlaboring parturients to be 0.35 IU [4]. These results suggest that doses lower than those commonly used would not only be effective, but would also decrease the risks of side effects [4–8].

When administered as an infusion, Lavoie et al. estimated the ED_90_ of oxytocin during elective CD in nonlaboring women to be 16.2 IU h^− 1^ [5]. While this approach is effective, it exposes patients to significant amounts of oxytocin in the postpartum period. Part of the reason that the ED_90_ is so high with this approach is because the infusion needs to be administered for some duration of time prior to achieving therapeutic plasma levels.

By administering an oxytocin bolus prior to initiation of infusion, therapeutic plasma concentrations are rapidly achieved and are simply maintained by the infusion [5]. Therefore, the aim of this study was to estimate the ED_95_ (the minimum effective dose (ED) of oxytocin bolus to result in adequate tone in 95% of parturients) for oxytocin infusion after a bolus at elective CD in nonlaboring parturients. Our hypothesis was that the ED_95_ of the infusion would be lower than in previous studies that did not administer a bolus prior to the infusion.

## Methods

The Ethical Committee of the Women’s Hospital, Zhejiang University School of Medicine (Hangzhou, China) approved this study on April 5, 2018 (No. 20180010). This study was registered on the Chinese Clinical Trial Register (ChiCTR1800015532).

The study was performed at the Women’s Hospital, Zhejiang University School of Medicine from April 9 to July 31, 2018. After providing written informed consent, 150 healthy term parturients scheduled for elective CD were enrolled in this prospective, randomized, triple-blinded, placebo-controlled, dose-finding study. The present study adhered to CONSORT guideline.

Inclusion criteria were American Society of Anaesthesiologists Physical Status II, age 18–40 years old, body mass index < 40 kg/m^2^, singleton pregnancy, ≥37 weeks’ gestation age, elective CD planned with a Pfannenstiel incision, and planning epidural anaesthesia. Parturients were only recruited if the individual performing the CD was 1 of 5 experienced obstetricians who agreed to have their patients participate in this study. Exclusion criteria included maternal refusal, emergency CD, active labor, ruptured membranes, pregnancy-induced hypertension, placental abnormalities (including placenta previa), multiple gestation, uterine fibroids, history of prior peripartum hemorrhage, coagulation disorders, oxytocin allergy, contraindication to epidural anaesthesia, and the need for pharmacological anxiolysis.

Prior to initiation of this study, sealed, opaque envelopes were prepared using the Microsoft Excel RAND function to determine study subject allocation. Participants were randomized to oxytocin infusion at a rate of 0, 1, 2, 3, 5, or 8 IU h^− 1^, to be given for a total of 1 h. On the day of surgery, an anaesthetist not involved in the study prepared an oxytocin infusion according to the randomization assignment. After filling a syringe with the total number of units of oxytocin that were to be administered during the hour-long infusion (e.g. if the patient were randomized to receive 8 IU h^− 1^, a total of 8 IU were drawn into the syringe), the syringe was filled with normal saline to make a total volume of 50 ml. This syringe was then given to the anaesthetist of record with instructions to administer the infusion at 50 ml over 1 h. By having all study subjects receive the same infusion rate (50 ml h^− 1^) but with a different total dose of oxytocin, the study subject, obstetrician, and anaesthetist responsible for the care of the patient were all blinded to the actual dose of oxytocin.

Upon entry into the operation room, an 18-gauge i.v. catheter was placed in the study subject’s lower forearm and 500 ml lactated Ringer’s solution was administered. With the parturient in the left lateral decubitus position, an epidural catheter was placed at the L_1–2_ interspace by an anaesthetist not involved in the study. Lidocaine 2% with epinephrine 1:200,000 was administered in 5 ml increments to a total of 15–20 ml until a T6 sensory level to pinprick was obtained.

Upon initiation of epidural anaesthesia, non-invasive blood pressure (NIBP) and maternal heart rate (HR) were measured at 3-min intervals. An i.v. bolus of ephedrine 5 mg was administrated when hypotension was accompanied by bradycardia (HR < 50 beats min^− 1^), and an i.v. bolus of phenylephrine 100 μg was administrated when hypotension occurred without bradycardia. Hypotension was defined as a decrease in systolic blood pressure greater than 20% from baseline, which had been previously estimated upon admission by the averaging of three consecutive measurements.

Surgery commenced with onset of adequate surgical anaesthesia. After clamping of the umbilical cord and delivery of the infant, all parturients were given an i.v. bolus of oxytocin 1 IU over 15 s as previously recommended [6], after which the oxytocin infusion previously prepared according to the randomization scheme was initiated at 50 ml h^− 1^.

After initiation of the oxytocin infusion, uterine tone (UT) was assessed by the obstetrician as adequate or inadequate every 3 min until the peritoneum was closed based on previously described methods [5–7]. If UT was assessed as inadequate, an i.v. bolus of oxytocin 1 U was administered. If UT was still judged to be inadequate after two such oxytocin boluses, then secondary uterotonic agents (i.m. carboprost tromethamine 0.25 mg or i.v. carbetocin 0.1 mg) were administered upon the obstetrician’s request. The oxytocin infusion was continued until discharge from the post anaesthetic care unit (PACU). If another syringe was needed in PACU, the same dose of oxytocin as the original was prepared.

The primary study outcome was adequacy of UT during the CD. Secondary outcomes included estimated blood loss (EBL), hemoglobin (Hb) and haematocrit (HCT) levels (at time of PACU discharge and on postoperative day 1), proportion of participants requiring administration of supplemental oxytocin boluses or alternative uterotonic agents, and side effects (hypotension, bradycardia, tachycardia [defined as HR ≥120 beats min^− 1^], nausea, vomiting, flushing, chest pain, or dyspnea). Postpartum hemorrhage (PPH), defined as EBL > 1000 ml [6], and the need for perioperative blood transfusion were also noted. EBL was estimated using the following formula [4, 9]: EBL (mL) = [(preoperative HCT - postoperative HCT)/preoperative HCT] × (weight in kilograms) × 85.

### Statistical analysis

Data are expressed as mean ± SD, median (inter-quartile range), or n (%) where appropriate. Data were assessed for normal distribution of variance using the Kolmogorov-Smirnov test. Normally distributed data were assessed by one-way analysis of variance. Nonnormally distributed data were assessed by the Kruskal-Wallis test. Categorical variables were assessed using the Chi-square test or Fisher exact test where appropriate. The Chi-square trend test (linear-by-linear association) was used to analyze the frequency of administration of additional uterotonic agents in the six groups. Statistical analyses were performed using SPSS version 16.0 (SPSS Inc., Chicago, IL, USA). *P* < 0.05 was considered statistically significant.

The dose-response relation for oxytocin infusion was determined using probit regression [10, 11]. An effective oxytocin infusion rate (success) was defined as a rate that provided adequate UT throughout the CD, from initiation of the oxytocin infusion to closure of the peritoneum, in the absence of administration of additional uterotonic agents. Data for successful responses for each infusion rate were used to plot a sigmoid dose-response curve. The ED_50_ and ED_95_ of an effective oxytocin infusion rate were then determined.

### Sample size calculation

The sample size was estimated using the Cochran-Armitage Test for the trend in proportions using PASS® (Version 11.0.7, NCSS, LLC, Kaysville, UT). Based on pilot data in which the proportion of parturients with adequate UT was 0.5, 0.5, 0.7, 0.8, 0.85, and 0.9 in those receiving oxytocin infusions at a rate of 0, 1, 2, 3, 5, or 8 IU h^− 1^, respectively, a total sample of 78 subjects (13 per group) were required to achieve 90% power to detect a liner trend using a two-sided Z test with continuity correction and a significance level of 0.05. We planned to recruit150 subjects to account for potential attrition.

## Results

**Fig. 1:**
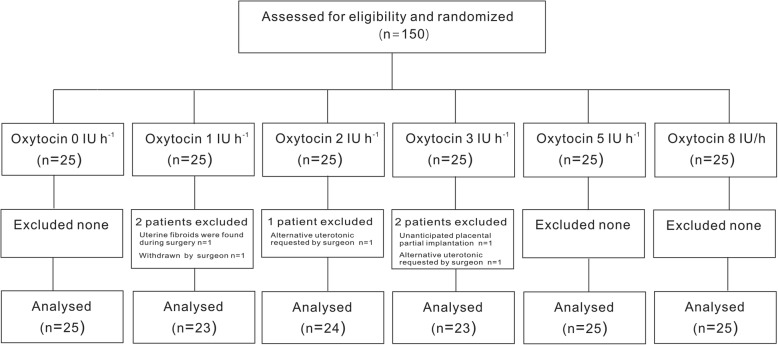
Study flow diagram. IU, units

Of a total 150 parturients who were enrolled, 145 completed the study (Fig. 1). There were no significant baseline differences among the six groups in demographic characteristics or preoperative Hb and HCT values (Table 1).

**Table 1:** Demographic and obstetric data

	0 IU h^− 1^	1 IU h^− 1^	2 IU h^− 1^	3 IU h^− 1^	5 IU h^− 1^	8 IU h^− 1^	*P* value
Age (yr)	34 ± 5	34 ± 5	32 ± 4	34 ± 4	32 ± 4	33 ± 4	0.108
Weight (kg)	69.1 ± 6.9	67.5 ± 7.1	68.3 ± 8.0	67.8 ± 7.3	70.6 ± 8.0	66.5 ± 6.7	0.463
Height (cm)	160.8 ± 3.8	159.6 ± 4.5	160.3 ± 3.9	158.7 ± 4.0	160.7 ± 5.1	159.9 ± 5.0	0.590
Prior CD	1 (0–1)	1 (0–1)	1 (0–1)	1 (0–1)	1 (0–1)	1 (0–1)	1.000
Gestational age (wk)	38 ± 1	38 ± 1	38 ± 2	38 ± 1	38 ± 1	38 ± 1	0.989
Duration of surgery (min)	38.8 ± 8.6	42.8 ± 9.7	40.5 ± 7.8	38.4 ± 8.9	39.2 ± 8.6	38.2 ± 9.1	0.479
Preoperative Hb (g dL^− 1^)	12.1 ± 0.8	12.2 ± 1.0	11.7 ± 1.3	12.1 ± 1.0	11.6 ± 1.1	12.1 ± 0.8	0.172
Preoperative HCT (%)	35.9 ± 2.3	35.8 ± 2.9	35.2 ± 3.4	35.7 ± 2.4	34.5 ± 2.5	36.1 ± 2.1	0.289

Data are expressed as mean ± SD, except prior CD which is expressed as median (inter-quartile range). *CD* Cesarean delivery, *Hb* hemoglobin, *HCT* hematocrit

**Fig. 2:**
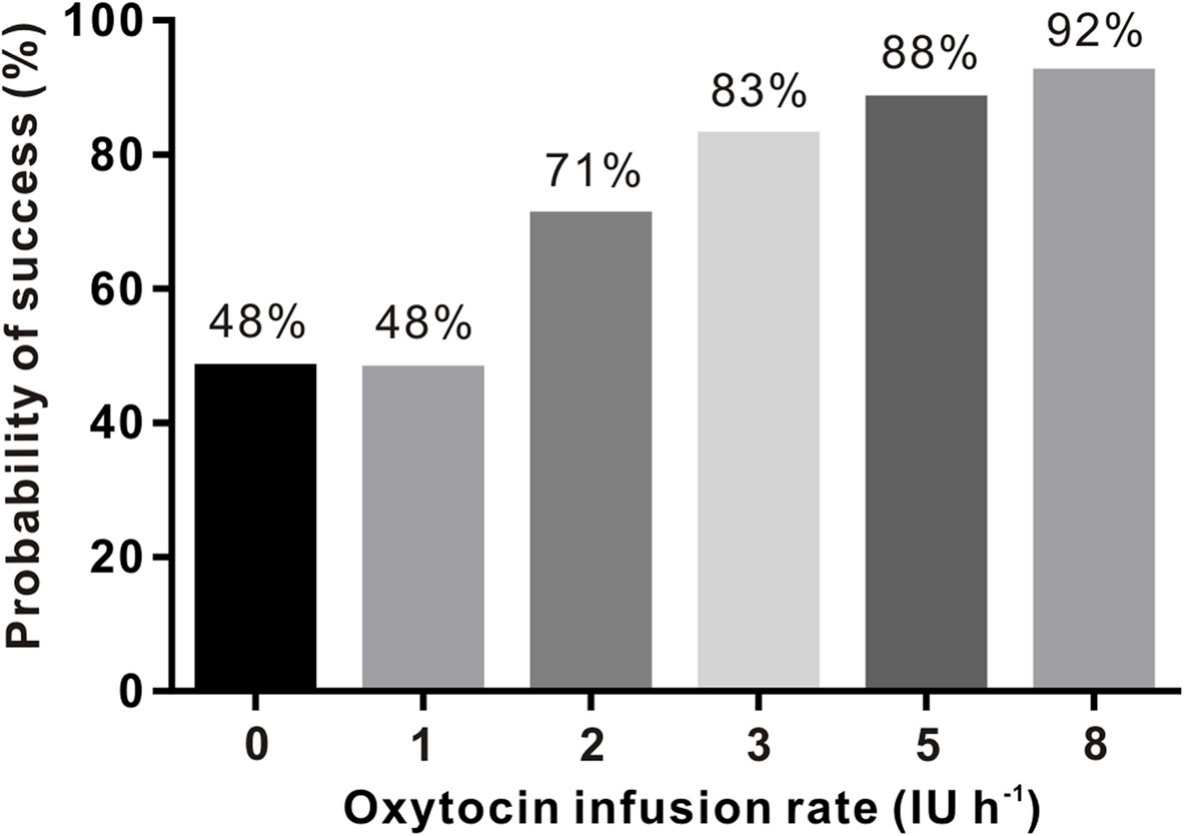
The proportion of study subjects with adequate uterine tone with different infusion rates of oxytocin

**Fig. 3:**
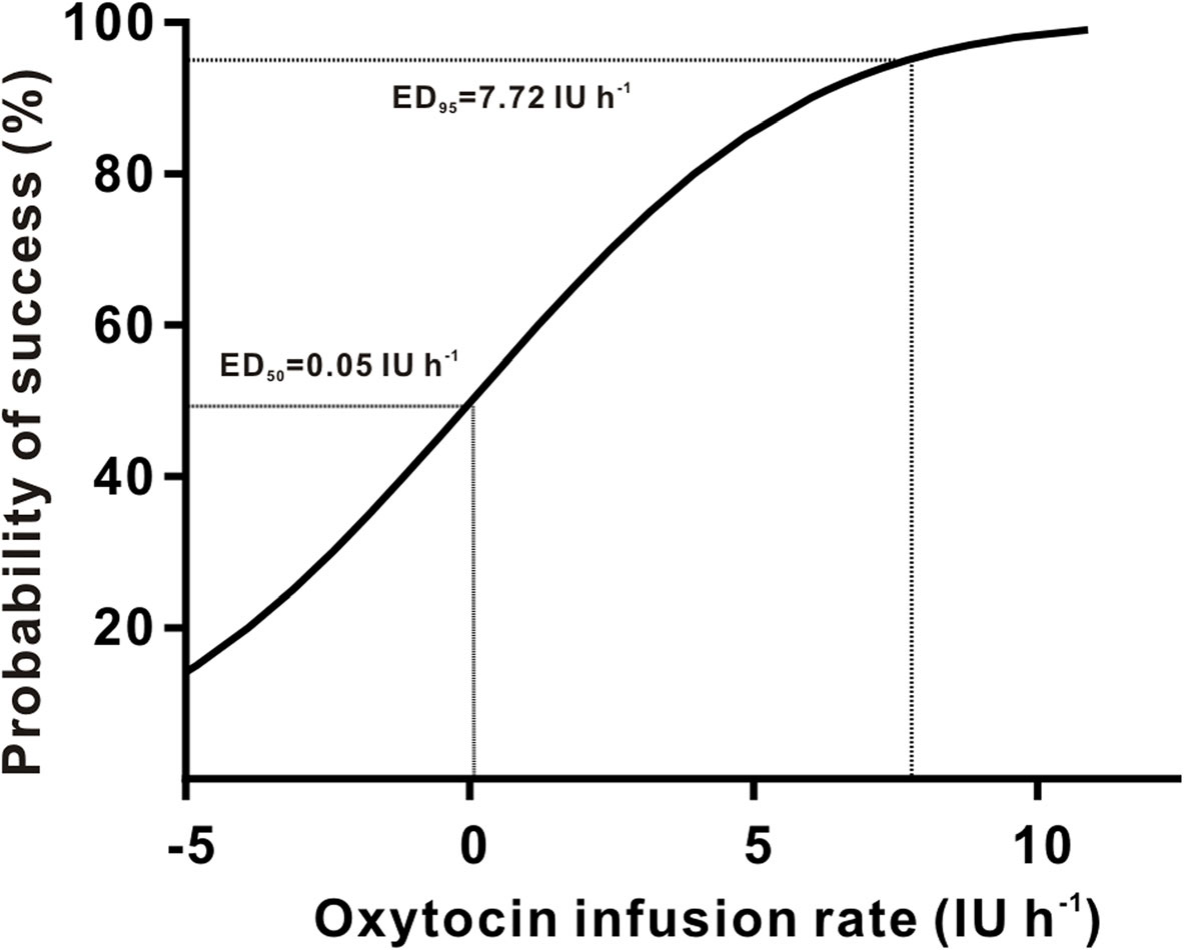
The ED_50_ and ED_95_ of oxytocin infusion. Regression plot of the probit value vs. the infusion rate of oxytocin. The 0.5 and 0.95 y-intercepts indicate the ED_50_ and ED_95_, respectively

The adequacy of UT during CD in the setting of each of the 6 randomized groups is summarized in Fig. 2. Using probit analysis, the dose-response curve was plotted (Fig. 3) from which we estimated the ED_50_ to be 0.05 IU h^− 1^ (95% confidence interval [CI] -2.51 to 1.25 IU h^− 1^) and the ED_95_ to be 7.72 IU h^− 1^ (95% CI 5.80 to 12.67 IU h^− 1^).

The results of the secondary outcomes are summarized in Table 2. The proportion of study subjects who required administration of supplemental oxytocin boluses or alternative uterotonic agents decreased with increasing oxytocin infusion rates. Compared with the placebo infusion group, fewer patients in the 3, 5, and 8 IU h^− 1^ oxytocin groups needed rescue oxytocin bolus (52% vs 17, 12, and 8%; *P* = 0.012, 0.002, and 0.001, respectively) or secondary uterotonic agents (24% vs 0, 0, and 0%; *P* = 0.023, 0.022, and 0.022, respectively). No significant differences in EBL and total i.v. crystalloid volume infused were observed. Hb and HCT levels measured at the time of PACU discharge and on postoperative day 1 were similar among the groups. There were no cases of PPH, postoperative Hb < 7 g dl^− 1^, or postoperative HCT < 24% observed. No parturients received blood transfusion perioperatively.

**Table 2:** Secondary outcomes

	0 IU h^− 1^	1 IU h^− 1^	2 IU h^− 1^	3 IU h^− 1^	5 IU h^− 1^	8 IU h^− 1^	*P* value
Estimated blood loss (ml)	693 ± 426	701 ± 394	619 ± 322	631 ± 404	625 ± 472	658 ± 330	0.977
Intravenous crystalloid (ml)	900 ± 210	993 ± 283	992 ± 273	1004 ± 296	1024 ± 255	984 ± 206	0.622
Hb in PACU (g dL^− 1^)	10.7 ± 1.0	10.8 ± 1.3	10.5 ± 0.9	10.9 ± 1.1	10.8 ± 1.2	10.7 ± 0.9	0.864
HCT in PACU (%)	32.4 ± 2.8	32.2 ± 3.8	31.7 ± 2.9	32.5 ± 3.1	31.9 ± 3.4	32.1 ± 2.5	0.956
Hb on postoperative day 1 (g dL^− 1^)	11.5 ± 1.1	11.3 ± 1.5	11.3 ± 1.1	11.8 ± 1.3	11.1 ± 1.1	11.6 ± 1.0	0.354
HCT on postoperative day 1 (%)	34.0 ± 3.0	33.3 ± 4.3	33.5 ± 3.0	34.7 ± 3.5	32.6 ± 2.9	34.2 ± 2.7	0.303
Rescue oxytocin required [n (%)]	13 (52)	12 (52)	7 (29)	4 (17) ^a^	3 (12)^b^	2 (8)^c^	0.000
Secondary uterotonic agents required [n (%)]	6 (24)	1 (4)	1 (4)	0 (0)^d^	0 (0)^e^	0 (0)^f^	0.000

Data are expressed as mean ± SD or n (%). Compared with 0 IU h–1 oxytocin infusion group, fewer patients in the 3, 5, and 8 IU h–1 oxytocin groups needed rescue oxytocin bolus (52% vs 17, 12, and 8%; ^a^*P* = 0.012, ^b^0.002, and ^c^0.001, respectively) or secondary uterotonic agents (24% vs 0, 0, and 0%; ^d^*P* = 0.023, ^e^0.022, and ^f^0.022, respectively). *PACU* postanesthetic care unit, *Hb* hemoglobin, *HCT* hematocrit

Side effects are shown in Table 3. We found no significant differences in the incidence of hypotension, tachycardia, nausea, flushing, and chest pain. Tachycardia was observed in one parturient who received the 8 IU h^− 1^ oxytocin infusion. No parturients experienced bradycardia, vomiting, or dyspnea during the study period.

**Table 3:** Oxytocin-related adverse effects

	0 U h^− 1^	1 U h^− 1^	2 U h^− 1^	3 U h^− 1^	5 U h^− 1^	8 U h^− 1^	*P* value
Hypotension	5 (20)	4 (17)	4 (17)	4 (17)	3 (12)	2 (8)	0.879
Tachycardia	0	0	0	0	0	1 (4)	1.000
Nausea	5 (20)	3 (13)	2 (8)	1 (4)	2 (8)	3 (13)	0.604
Flushing	5 (20)	6 (26)	5 (21)	6 (26)	5 (20)	5 (20)	0.986
Chest pain	4 (16)	2 (9)	1 (4)	1 (4)	1 (4)	2 (8)	0.582

Data are expressed as n (%)

## Discussion

The primary finding of this dose-response study was that, in nonlaboring women undergoing elective CD, the ED_50_ of an oxytocin infusion was 0.05 IU h^− 1^ (95% CI − 2.51 to 1.25 IU h^− 1^) and the ED_95_ was 7.72 IU h^− 1^ (95% CI 5.80 to 12.67 IU h^− 1^). In addition, fewer parturients in 3, 5, and 8 IU h^− 1^ oxytocin infusion groups required rescue oxytocin bolus or secondary uterotonic agents compared with placebo. Finally, no significant differences in the EBL or the incidence of oxytocin-related side effects were observed among groups.

The ED_90_ of oxytocin as an infusion (without a pre-infusion bolus) during elective CD has been previously estimated to be between 16.2 IU h^–1 5^ and 17.4 IU h^− 1^ [12]. We found that when a pre-infusion bolus is administered, the ED_95_ of an oxytocin infusion is reduced to 7.72 IU h^− 1^. The finding that the ED_95_ of an oxytocin infusion in the setting of a pre-infusion bolus is lower than without is clinically significant because it suggests that lower doses overall may be needed when administered in this fashion. By administering lower doses, the risk for side effects would presumably also be decreased, thus improving the care of this patient population.

The finding that the ED_95_ of an oxytocin infusion in the setting of a pre-infusion bolus is lower than without is not entirely surprising. The basic principles of pharmacokinetics dictate that bolus administration of oxytocin results in rapid attainment of therapeutic plasma concentrations [5]. Therefore, the purpose of the infusion in this setting is simply to maintain those levels after the initial bolus. On the other hand, when an infusion is started without a bolus, there will be a delay until therapeutic plasma concentrations are attained, which may increase the risk for uterine atony.

Our study is consistent with prior study which emphasize that a bolus of oxytocin alone is insufficient [9]. When compared to the placebo group (no oxytocin infusion), those who received an infusion of 3, 5, and 8 IU h^− 1^ required fewer rescue oxytocin boluses or secondary uterotonic agents. This finding is not surprising because oxytocin has an extremely short half-life (4–10 min) [9], so in order to maintain UT after the initial bolus, an infusion would be absolutely necessary.

While our study findings have important clinical implications, there are several limitations that should be considered. First, given that assessment of adequate UT was done subjectively, it is unknown if similar results might be obtained if a different set of obstetricians made the assessment. However, given that Lavoie et al. [5] and George et al. [12] obtained similar results using similar methods, it is likely that our results would be similar even if UT were to be assessed by different providers. Second, because we studied only nonlaboring parturients undergoing elective CD, it is likely that the ED_95_ would be different if studying in a different population. Finally, while we chose to administer a bolus of 1 IU because it is well above the ED_90_ oxytocin [4], a smaller or larger bolus could have altered the ED_95_. Further studies should be performed to determine if a similarly low ED_95_ of oxytocin infusion is found if a lower bolus dose of oxytocin is used.

## Conclusions

This study demonstrates that the ED_95_ of oxytocin infusion is decreased when a small bolus of oxytocin is administered prior the infusion. This finding is important because excessive oxytocin administration is associated with side effects, including nausea, vomiting, and hypotension. Future studies should be performed to better understand if a smaller dose of the bolus could be used and still result in an overall decreased need for oxytocin infusion.

## Data Availability

All data generated or analysed during this study are included in this published article.

## Abbreviations

CD: Caesarean delivery
EBL: Estimated blood loss
ED: Effective dose
Hb: Haemoglobin
HCT: Haematocrit
HR: Heart rate
NIBP: Non-invasive blood pressure
PACU: Post anaesthetic care unit
PPH: Postpartum haemorrhage
UT: Uterine tone
